# Diffuse Adenomyomatosis of Gallbladder: A Rare Case Report With Insights Into a Distinctive Condition

**DOI:** 10.7759/cureus.72314

**Published:** 2024-10-24

**Authors:** Shobana B., Divya Lakshmi, Mary Lilly S., Shivangi J Shindhe

**Affiliations:** 1 Department of Pathology, Sree Balaji Medical College and Hospital, Chennai, IND

**Keywords:** adenomyoma, adenomyomatosis, asymptomatic, benign, gall bladder

## Abstract

Gallbladder adenomyoma/adenomyomatous nodule/adenomyomatosis is a benign condition characterized by mucosal invaginations within the gallbladder wall with or without proliferation of smooth muscle fibers. Gallbladder adenomyoma is often asymptomatic and discovered incidentally during imaging studies performed for other unrelated conditions. It can mimic malignancy on radiological findings, creating a diagnostic dilemma. The management and outcome of adenomyoma and malignant lesions of gallbladder are completely different. We present here a rare case of 52-year-old female diagnosed with gallbladder adenomyoma in histopathological examination highlighting the insights into this benign distinctive condition. Therefore, it is essential to completely understand the pathognomonic and histopathological features of gallbladder adenomyoma in order to accurately diagnose this condition.

## Introduction

Gallbladder adenomyoma/adenomyomatous nodule is a relatively rare but benign condition characterized by mucosal invaginations within the gallbladder wall with proliferation of smooth muscle fibers [1,2].

Diverticular abnormalities called Aschoff-Rokitansky (RA) sinuses are common in chronic cholecystitis. Some authors believe that adenomyomas are exaggerated phenomenon of RA sinuses [1,2]. RA sinuses may sometimes exhibit reactive epithelial atypia, which can be extremely severe and misinterpreted for invasive cancer. Conversely, cholelithiasis and cholecystitis are frequently associated with gallbladder carcinomas, which can have a deceptively innocuous appearance [1].

Prevalence of adenomyoma in cholecystectomy specimens is estimated between 1% to 9% and the incidence increases after the age of 50 [3]. Studies show different incidences for men and women, but some publications also claim similar incidences [3].

Regardless of whether these adenomyomatous nodules in gallbladder, a sign of persistent damage or just an amplification of RA sinuses or a developmental defect, the etiology and pathogenesis of this condition are still not well understood [2,4].

This condition has unique histological features; understanding its nature and differentiating it from other gallbladder lesions especially adenocarcinoma is crucial for its diagnosis and proper management.

This case report explores the key aspects of gallbladder adenomyoma, providing a comprehensive overview of the various terminologies used, its clinical significance, diagnostic histopathological features, and appropriate management strategies.

## Case presentation

**Figure 1:**
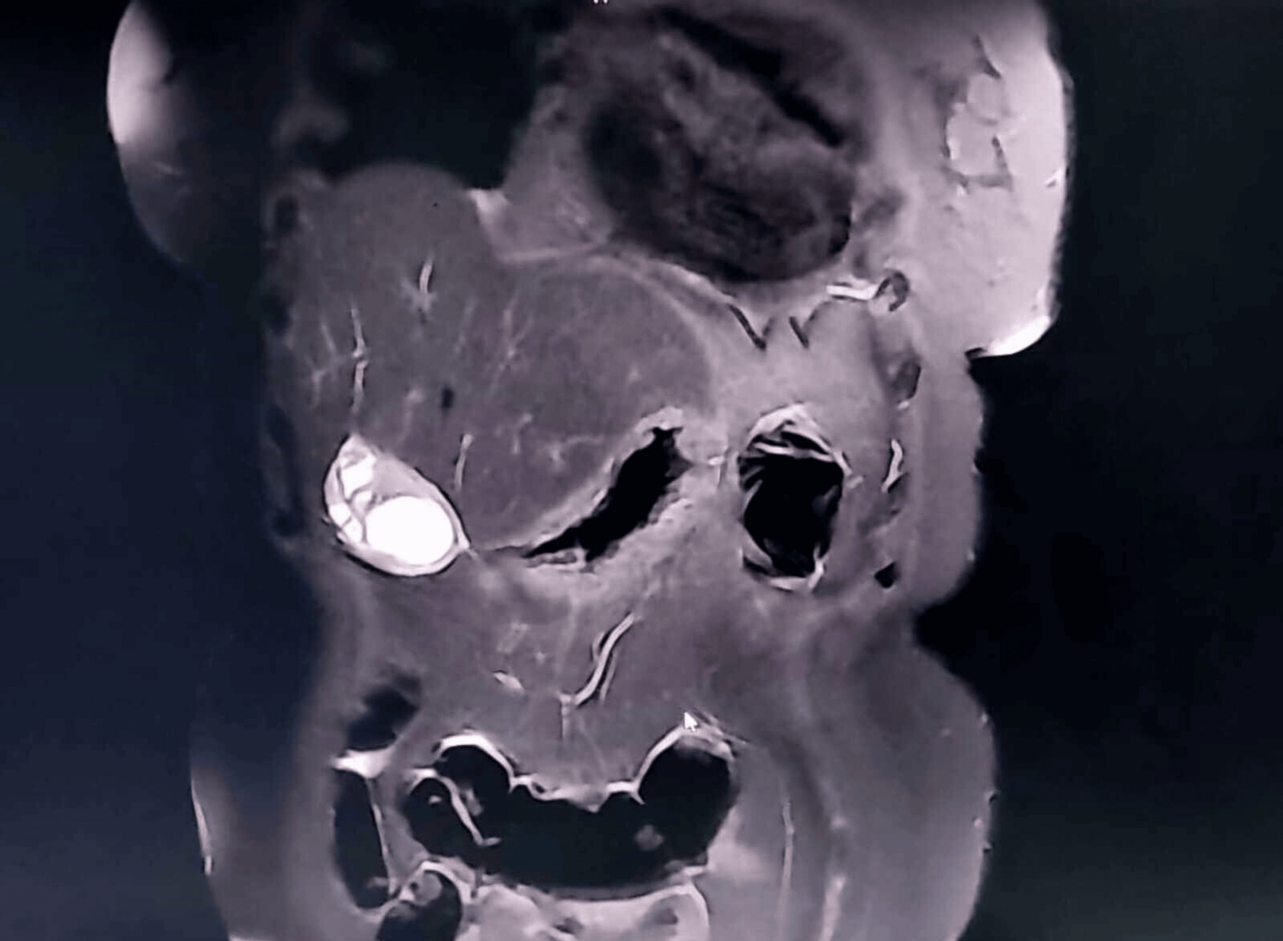
T2 coronal section of MRCP Distended gallbladder with thickened gallbladder wall seen involving the body and fundus.

**Figure 2:**
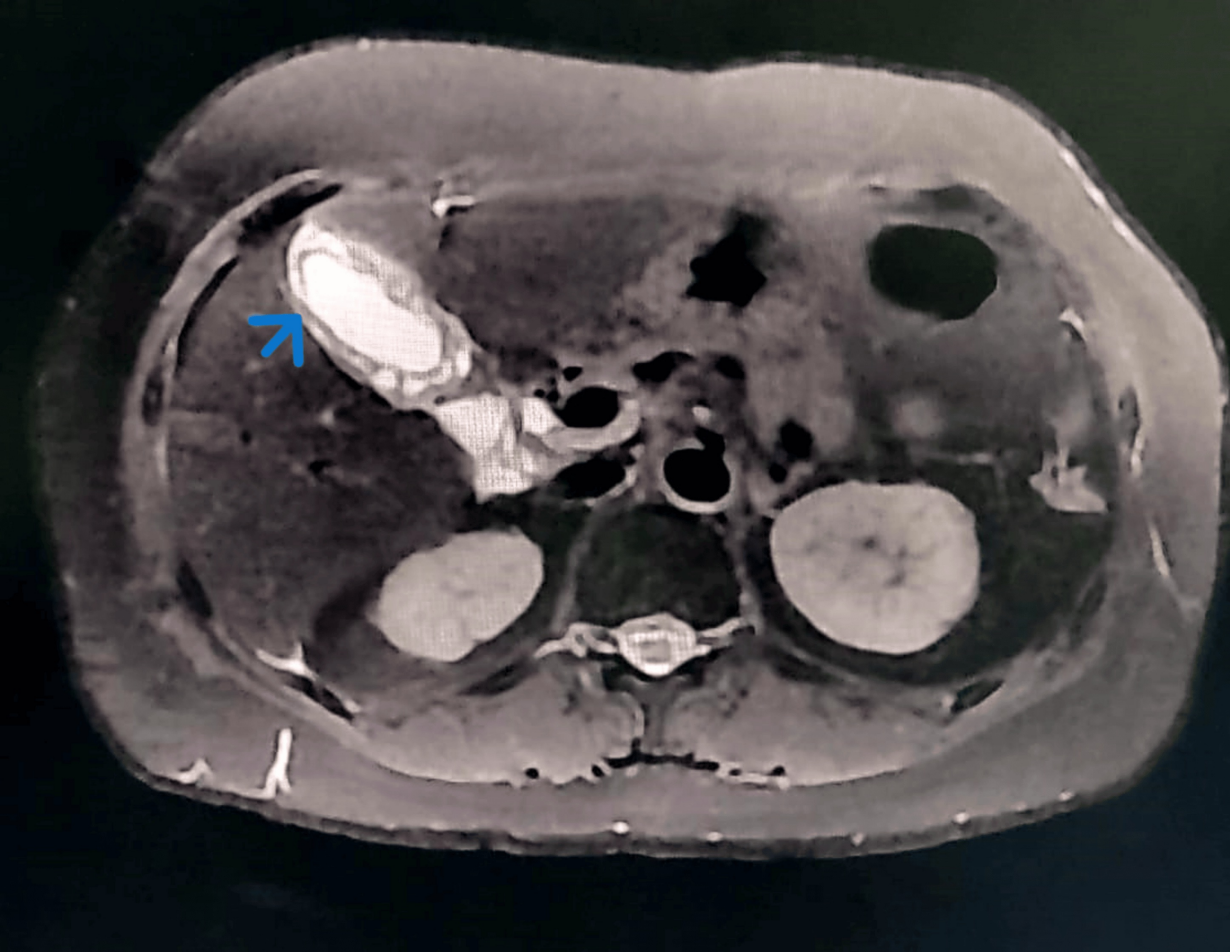
T2 axial section of MRCP Diffuse circumferential gallbladder wall thickening seen involving the body and fundus with multiple cystic spaces (blue arrow) and restricted diffusion.

A 52-year-old female came to the OPD with complaints of intermittently recurring upper abdominal pain for one year. She also complained of vomiting for two weeks. Clinical diagnosis was given as cholecystitis with cholelithiasis. USG abdomen revealed calculous cholecystitis and grade 1 fatty liver. MRCP revealed diffuse circumferential gallbladder wall thickening involving the body of the gallbladder with multiple cystic spaces showing restricted diffusion and cholelithiasis (Figure 1, Figure 2).

Cholecystectomy was done and sent for histopathological examination. Gross examination revealed cholecystectomy specimen measuring 10x5.5x2 cm. External surface shows irregularly dilated and distended gallbladder.

**Figure 3:**
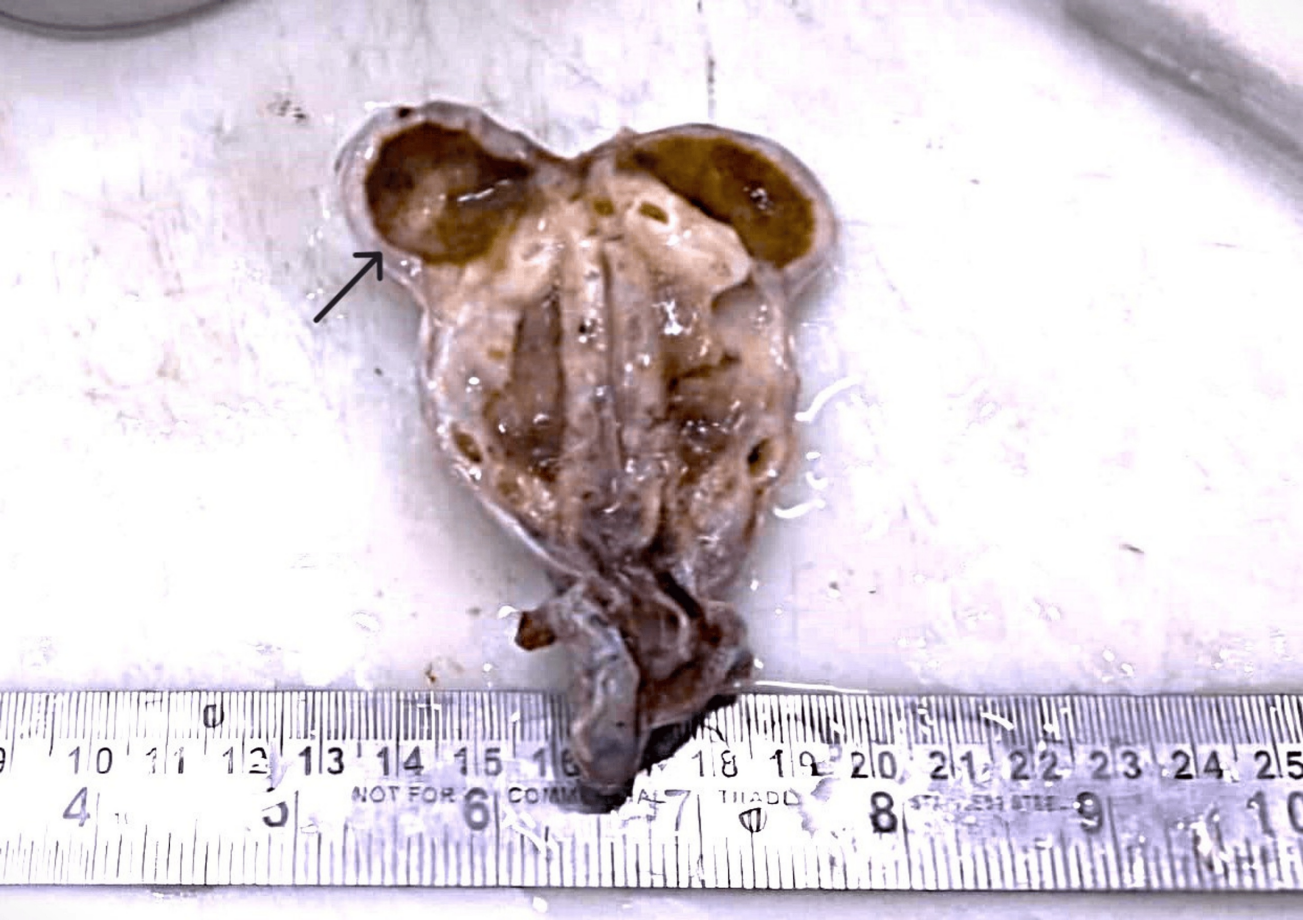
Gross specimen Markedly thickened gallbladder wall with multiple dilated cystic spaces. Note the large adenomyoma (near the fundus) (black arrow).

Cut surface shows thickened gallbladder wall with multiple small cystic spaces. A large cystic space is seen near the fundus (Figure 3). Three grey, yellow stones are noted. Gallbladder mucosa is velvety and bile stained. Multiple representative bits were taken.

**Figure 4:**
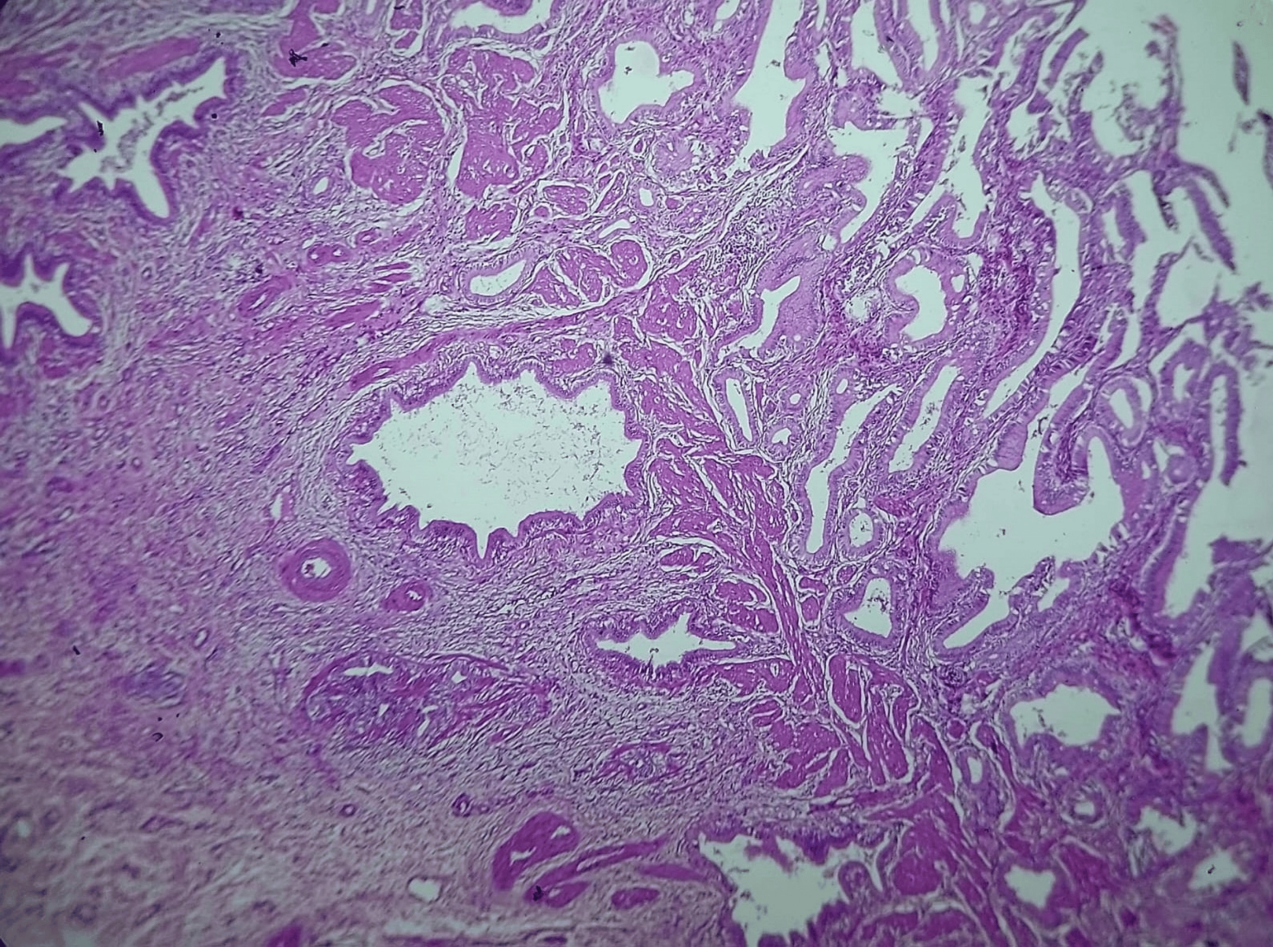
Low power view (10x, H&E stain) Hyperplastic mucosa with epithelial-lined glands in the subserosa and muscle layer.

**Figure 5:**
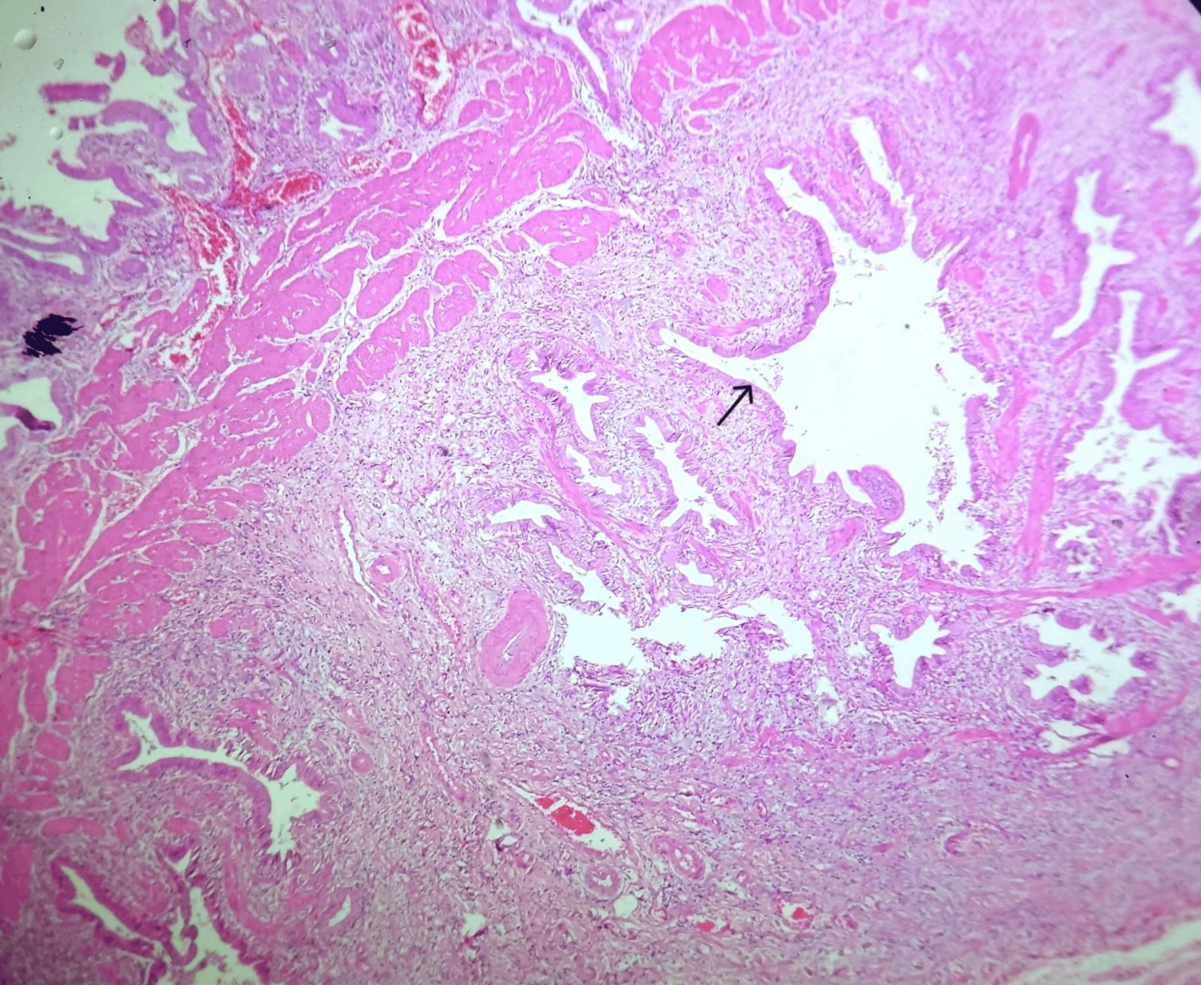
Low power view of fundic adenomyoma (10x, H&E stain) Multiple, irregular, dilated, and branching glands (black arrow) with prominent smooth muscle extending deeply into the gallbladder wall.

**Figure 6:**
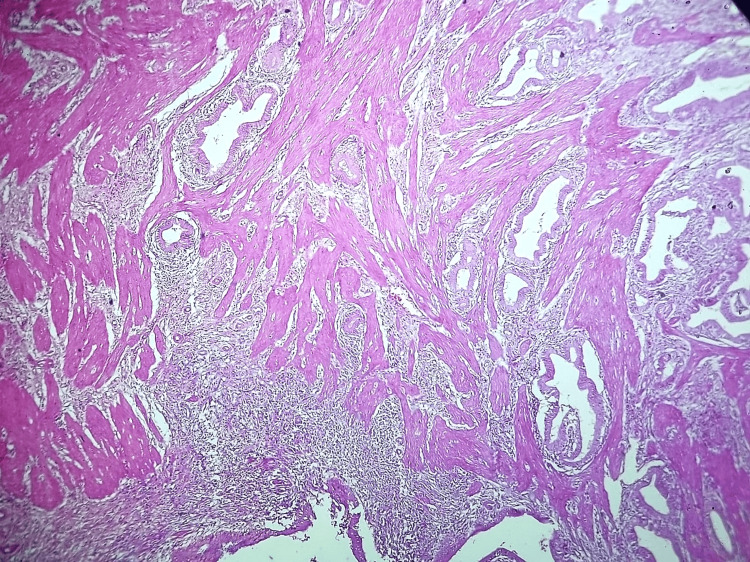
Low power view (10x, H&E stain) Hyperplastic muscle with chronic inflammation and haphazardly arranged glandular structures.

**Figure 7:**
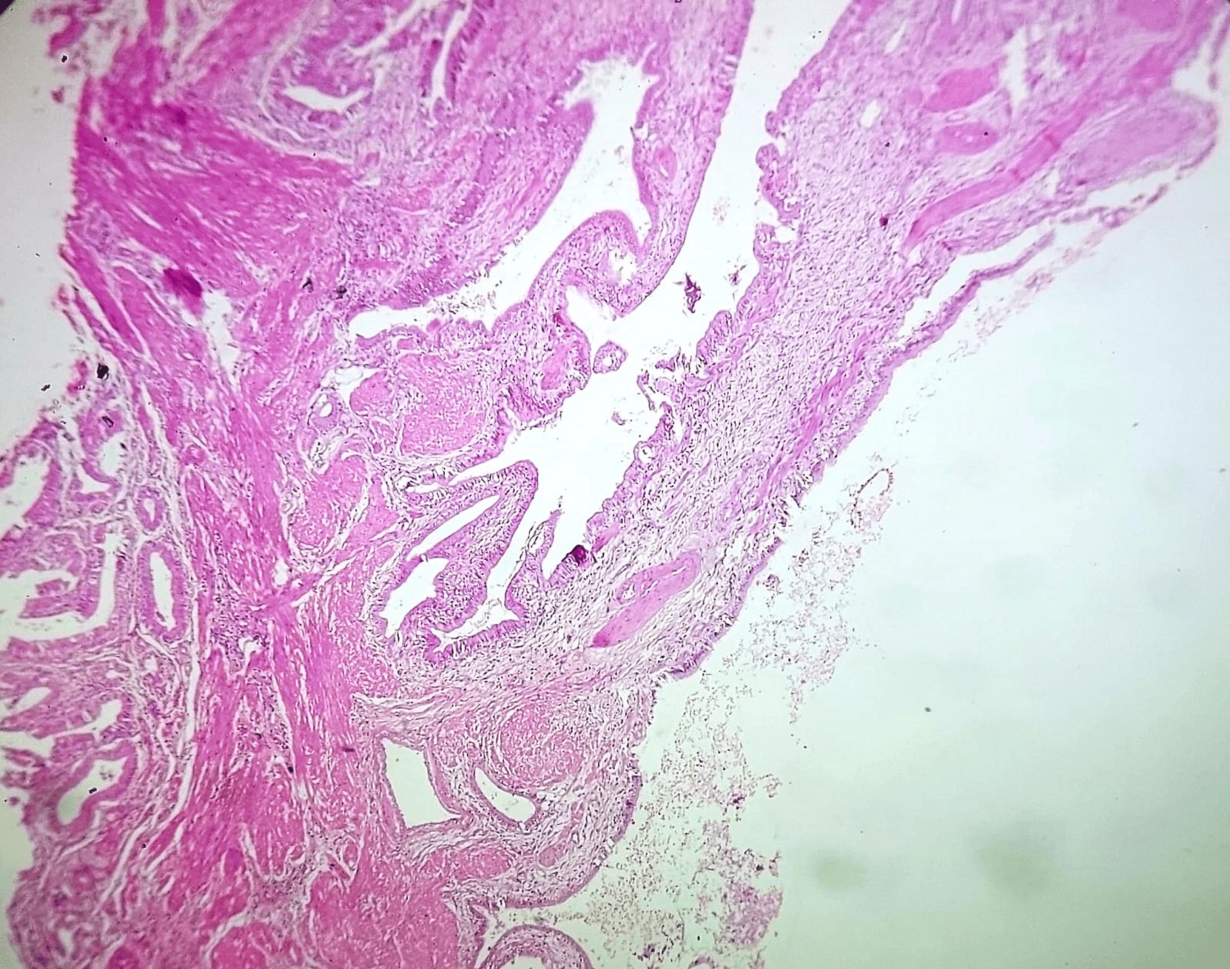
Low power view (10x, H&E stain) Diffuse adenomyomatosis extensively involving gallbladder wall.

**Figure 8:**
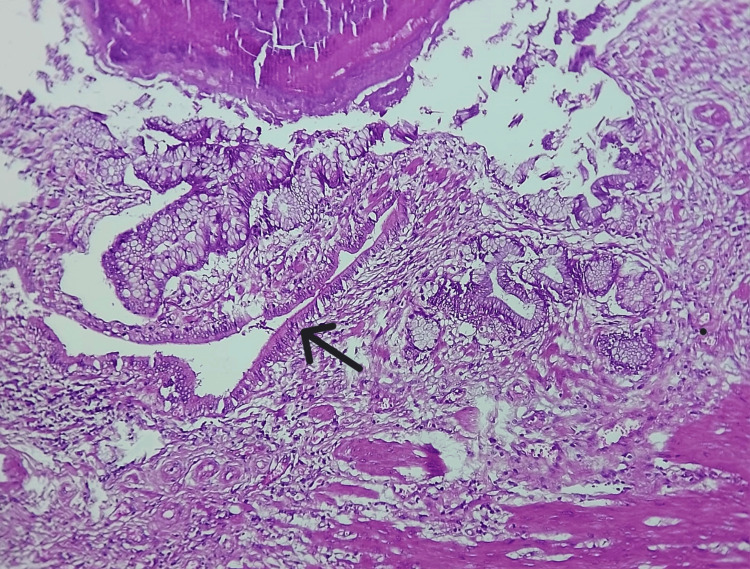
High power view of microlith and pyloric gland metaplasia in the adenomyoma (40x, H&E stain) Note the benign epithelium lining the glands without cyto-nuclear atypia (black arrow).

Microscopic examination revealed features of chronic calculous cholecystitis with hyperplastic mucosa (Figure 4), hyperplastic muscle bundles with entrapped diffuse, and dilated and irregular glandular structures lined by columnar epithelium (Figures 5, 6, 7). Tiny islands of pyloric gland metaplasia is also noted (Figure 8).

The final impression was reported as adenomyoma (diffuse adenomyomatosis) of the gallbladder with chronic calculous cholecystitis. Post-operatively, the patient recovered from surgery with appropriate antibiotics. Patient is on regular follow-up and doing good till now.

## Discussion

Adenomyoma or adenomyomatous nodules is characterized by cluster of cystically dilated glands in the gallbladder wall that are occasionally combined with prominent muscles. Studies show that the occurrence in males and women varies [3,5]. Most frequently, they are seen in the fundic area [2,6]. They create a tiny, isolated mass or a band of trabeculated thickening of the gallbladder wall [2]. The more widespread, extremely rare variant of this condition is referred to as "adenomyomatosis" as in our case, which is a very rare occurrence (Figure 4). 

Terminology and controversies

Various terms (hyperplastic adenomyosis, adenofibromyoma, and proliferative glandular sickness) were used to refer to this proliferative condition until the 1960s till Judas simplified the phrase and gave it the label gallbladder adenomyomatosis [3].

While some believe it to be an exaggerated version of RA sinuses, in many cases, there is no communication with the surface mucosa; no additional sinuses are present. Furthermore, even though the term suggests a myoid process, the muscular component is sometimes absent or very little. However, our case has myoid component and associated chronic cholecystitis and cholelithiasis. Some experts argue to call them adenomyomatous nodules since they are not real neoplasms [7]. Some authors suggest that adenomyomatous hyperplasia, adenomyoma (when localized), and adenomyomatosis (when diffuse) are impressive but rather erroneous terms that have been used to denigrate exaggerated cases of gallbladder diverticulosis. The term adenomyomatous hyperplasia does not indicate true epithelial hyperplasia but it may harbor dysplasia within it [2,4]. Our case also showed epithelial hyperplasia focally but no dysplasia is seen in our instance.

Etiology and pathogenesis

The exact cause of gallbladder adenomyoma remains largely unknown. However, several theories suggest that smooth muscle hyperplasia and mucosal invaginations are believed to be adaptive responses resulting from chronic irritation or inflammation of the gallbladder wall [1]. It was initially recognized as a precancerous lesion but recent studies consider it as a benign alteration [7]. Correlation with acquired wall motility as a consequence of increased endoluminal pressure due to cholelithiasis has also been postulated, and further research is needed to fully understand the pathogenesis of this condition [4]. Gallstones and persistent cholecystitis have been linked in our case, corroborating these findings.

Clinical presentation

Gallbladder adenomyoma is often asymptomatic and discovered incidentally during imaging studies [8]. When symptoms do appear, they are usually nonspecific, as in our case and may include abdominal discomfort [6,9]. Furthermore, adenomyoma is typically found during histological evaluation of the samples of individuals undergoing surgery for symptomatic cholelithiasis. Thus, the clinical presentation of this condition might also be vague and misleading, as Teelucksingh et al. explain, and may only manifest as persistent discomfort [9].

Imaging studies

Ultrasound (US, high resolution) is the preferred imaging modality for preoperative diagnosis. There are three known morphological types: 1. Segmental: they divide the gallbladder body into two parts in the form of rings (as seen in our instance); 2. Fundal: present with local thickening; 3. Diffuse type: diffuse irregularly thickened gallbladder wall [3]. In our case, both the diffuse (a very unusual appearance) and segmental forms (found in the fundus) are present. In cases where the diagnosis is uncertain, MRI (cholangio MRI) is necessary [3]. The "pearl necklace" sign shown in MRCP was regarded as pathognomonic for gallbladder adenomyomatosis and its sensitivity is 80%, in a research done by Haradome et al. [7,10].

Histopathology

Grossly, adenomyomatous nodules might result in tiny cysts or trabeculations that resembles a sieve [1]. This can present as polypoid lesion as well [4,11]. A sharply confined lesion that can resemble an adenocarcinoma both radiographically and grossly is seen in localized type, which can affect any part but is typically seen in the fundus [6]. Often, cysts in the gallbladder wall can also arise from the Luschka ducts and tubulo-cystic variety of biliary adenocarcinoma; microscopic examination is definitely required in order to rule out these gross differential diagnoses. The lesion in microscopy consists of cystically dilated glands, within hypertrophic smooth muscle (Figures 4, 5); the epithelium may show metaplastic or reactive changes, similar to our case and these changes are seen in the entire wall of the gallbladder (diffuse adenomyomatosis) (Figure 8).

Differentiation from neoplastic process

In addition to these lesions simulating adenocarcinomas due to their pseudo invasive appearance, in situ or invasive carcinoma may be associated with them [1,5]. GA is not regarded as a premalignant lesion as it lacks intrinsic malignant potential and has no extrahepatic spread [3,5,12]. Many research have observed that carcinomas may occasionally be limited only to the adenomyomatous lesions [2]. In certain situations, GA may mimic intraductal papillary mucinous neoplasm if they exhibit widespread dysplastic changes and papillary structures [13].

Perineural invasion

It should be mentioned that glandular components of adenomyomatous nodules impinge on the nerves and resemble intraneural and perineural invasion [14]. Thus, the gallbladder is now one more organ in which this phenomenon can occur in the absence of malignancy [7].

Management

Cholecystectomy is reserved for symptomatic patients or inconclusive imaging findings [1,6]. Histopathological examination still remains the gold standard method for its precise diagnosis as in many situations imaging studies alone cannot rule out an associated malignancy [6]. Frozen section should be done during surgery in order to rule out gallbladder cancer (GC) in doubtful cases [15].

## Conclusions

Gallbladder adenomyoma/adenomyomatosis is a benign condition that is usually misdiagnosed both clinically and radiologically. Its radiological results and clinical presentation are deceptive. For proper patient care, it is crucial to distinguish between an adenomyoma and an early GC. Our rare case report presents this unique entity's insights with decision-making algorithm and emphasizes the need for histopathological evaluation. However, continued research and advancements in imaging technologies will definitely enhance the understanding and management of this unique gallbladder condition.
